# Dysregulation of Key Proteins Associated with Sperm Motility and Fertility Potential in Cancer Patients

**DOI:** 10.3390/ijms21186754

**Published:** 2020-09-15

**Authors:** Manesh Kumar Panner Selvam, Renata Finelli, Saradha Baskaran, Ashok Agarwal

**Affiliations:** American Center for Reproductive Medicine, Cleveland Clinic, Cleveland, OH 44195, USA; pannerm@ccf.org (M.K.P.S.); finelli.renata@gmail.com (R.F.); saradhabaskaran@gmail.com (S.B.)

**Keywords:** bioinformatics, cancer, male infertility, proteomics, sperm

## Abstract

Cancer has adverse effects on male reproductive health. Conventional semen analysis does not explain the molecular changes in the spermatozoa of cancer patients. Currently, proteomics is being widely used to identify the fertility-associated molecular pathways affected in spermatozoa. The objective of this study was to evaluate the sperm proteome of patients with various types of cancer. Cryopreserved semen samples from patients (testicular cancer, *n* = 40; Hodgkin’s disease, *n* = 32; lymphoma, *n* = 20; leukemia, *n* = 17) before starting therapy were used for proteomic analysis, while samples from fertile donors (*n* = 19) were included as controls. The proteomic profiling of sperm was carried out by liquid chromatography-tandem mass spectrometry, and differentially expressed proteins involved in the reproductive processes were validated by Western blotting. Bioinformatic analysis revealed that proteins associated with mitochondrial dysfunction, oxidative phosphorylation, and Sirtuin signaling pathways were dysregulated in cancer patients, while oxidative phosphorylation and tricarboxylic acid cycle were predicted to be deactivated. Furthermore, the analysis revealed dysregulation of key proteins associated with sperm fertility potential and motility (NADH:Ubiquinone oxidoreductase core subunit S1, superoxide dismutase 1, SERPINA5, and cytochrome b-c1 complex subunit 2) in the cancer group, which were further validated by Western blot. Dysfunctional molecular mechanisms essential for fertility in cancer patients prior to therapy highlight the potential impact of cancer phenotype on male fertility.

## 1. Introduction

Cancer in reproductive age can significantly affect fertility in males, representing one of the most common causes of infertility. The majority of people affected by cancer are >55 years old (~80%). In 2020, a high number of people in reproductive age (15–39 years old) are predicted to be affected by testicular cancer (6500 estimated new cases), Hodgkin’s lymphoma (2800 estimated new cases) and non-Hodgkin’s lymphoma (4600 estimated new cases), as well as leukemia (600 estimated new cases) in the United States [1]. As reported by the American Cancer Society, an increase in relative survival rate has been reported for all types of cancers since the 1960s. This may be due to early diagnosis and the availability of more effective treatments [1]. Hence, the possibility of achieving a future pregnancy is a concern for cancer patients [2].

Cancer therapies have gonadotoxic effects, which result in reduced semen quality and reproductive potential [3,4]. Moreover, the exposure to radiation can also have teratogenic impact as it increases the rate of sperm DNA mutation and the risk of genetic abnormalities in the offspring [5]. These therapies include radio- and chemotherapy, while surgery may be the final option [6]. Hence, the cryopreservation of semen sample is the only option for cancer patients before starting a gonadotoxic therapy to father a biological child in the future. However, besides the impact of gonadotoxic treatments on semen quality, preliminary studies report abnormal semen parameters, particularly sperm motility, in pre-treated cancer patients [7,8,9,10]. A recent retrospective study conducted by Auger et al., involving 4480 male patients of reproductive age affected by several types of cancer and systematic disease, reported a significant decrease in sperm concentration, progressive motility and normal morphology [10]. Specifically, testicular cancer and leukemia patients showed poor semen quality, as normozoospermia was observed only in 50.9% and 36.9% of patients, respectively [10]. Similar observations were also reported by other investigators [11,12,13,14]. Low sperm quality was observed in patients with Hodgkin’s disease when compared to non-Hodgkin’s lymphoma [15], while oligospermia was reported in more than 50% of the leukemia patients [16]. However, a recent study reported that 75% of patients affected by Hodgkin’s disease are normozoospermic prior to treatment [17]. In fact, better semen quality is observed in patients with Hodgkin’s disease than in those with testicular cancer [8,18].

Routine semen analysis performed in the workup of male infertility provides information regarding the quality of sperm, however it does not help elucidate the molecular mechanisms associated with infertility. The advent of proteomics has enabled to decipher the molecular mechanisms as well as the subcellular changes related to the fertilization potential of spermatozoa [19,20]. Hitherto, few proteomics studies have been conducted on semen samples of patients affected by specific types of cancer [21,22,23,24]. However, the general impact of cancer on protein expression in sperm has not been elucidated yet. Understanding the cellular and molecular mechanisms of cancer-associated male infertility is not only crucial for the management of male infertility, but also useful for fertility preservation in cancer patients. Proteomic results could (1) explain the pathways negatively affecting male fertility in pre-treatment patients, (2) help in achieving a better cryopreservation outcome in samples from cancer patients, (3) identify protein biomarkers for cancer-associated infertility and may (4) lead to more effective diagnosis and possible treatment of complications associated with cancer. Therefore, the main objective of this study was to investigate the sperm proteome of cancer patients before initiating cancer therapy in comparison with that of healthy fertile men.

## 2. Results

### 2.1. Semen Parameters

Sperm concentration and motility are reported in Table 1 for each group. Although the average values were within the physiological limits provided by the WHO guidelines, a significant reduction of sperm concentration was observed in all cancer groups, while a significant decline in sperm motility was noted in patients with testicular cancer and lymphoma when compared to the fertile donors.

### 2.2. Protein Profile of Cancer Patients

**Figure 1:**
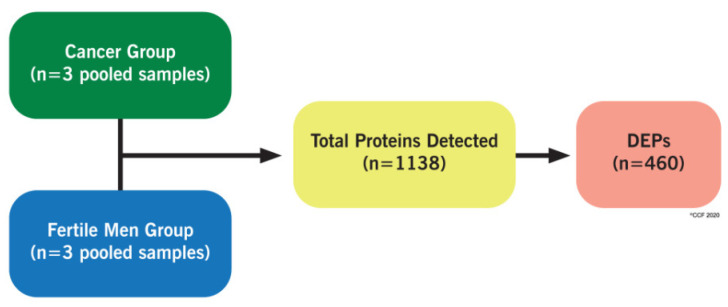
Proteome profile of sperm in cancer group and fertile men. DEPs: differentially expressed proteins.

Proteomic profiling of spermatozoa in cancer patients and fertile men revealed a total of 1138 proteins common to cancer group and fertile men, with 460 differentially expressed proteins (DEPs) (Figure 1).

**Figure 2:**
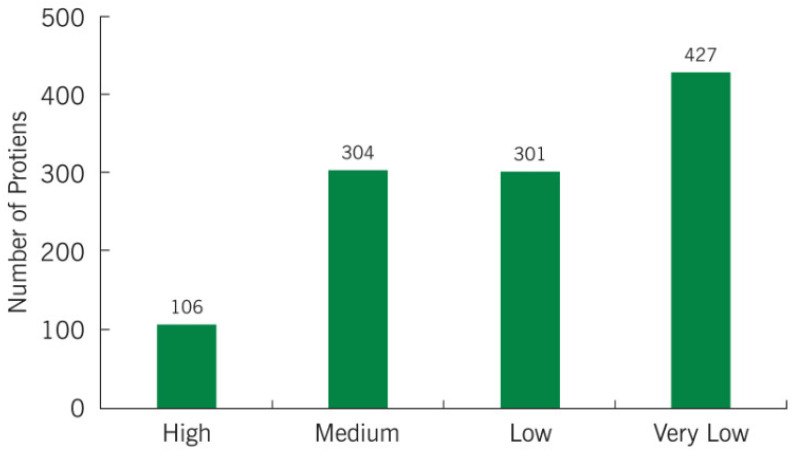
Abundance of sperm proteins identified in cancer/fertile groups.

A comparative analysis showed that 208 and 62 proteins were under- and over-expressed, respectively, in cancer group compared to fertile men, while 182 and 8 proteins were found to be uniquely expressed in fertile men and cancer patients, respectively. Table 2 provides the list of uniquely expressed proteins in spermatozoa of cancer patients when compared to fertile men. The abundance of the proteins expressed in the cancer group and fertile men are presented in Figure 2. Most of the proteins expressed in both groups were present in very low abundance (Figure 2).

### 2.3. Canonical Pathways

**Figure 3:**
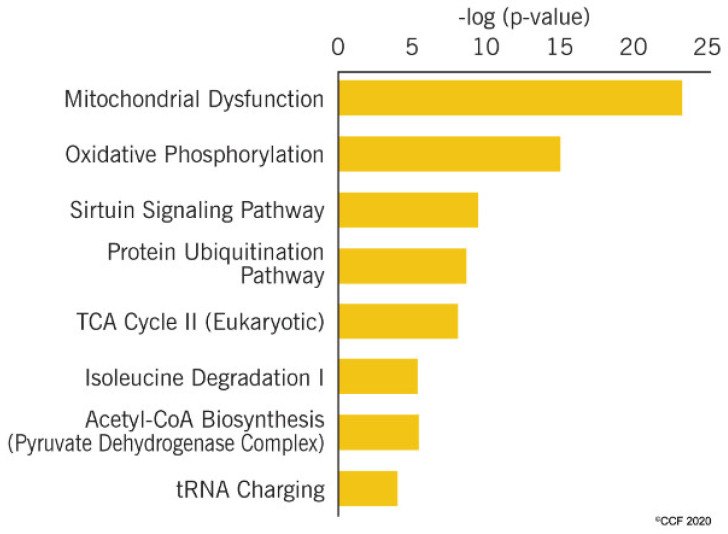
Higher -log (*p*-value) indicates the dysfunction of the corresponding pathways due to the involvement of differential expressed proteins. The figure shows that mitochondrial dysfunction, oxidative phosphorylation and Sirtuin signaling pathway, protein ubiquitination pathway, tricarboxylic acid (TCA) cycle II, isoleucine degradation I, acetyl-CoA biosynthesis and tRNA charging are the top canonical pathways affected in cancer group.

**Figure 4:**
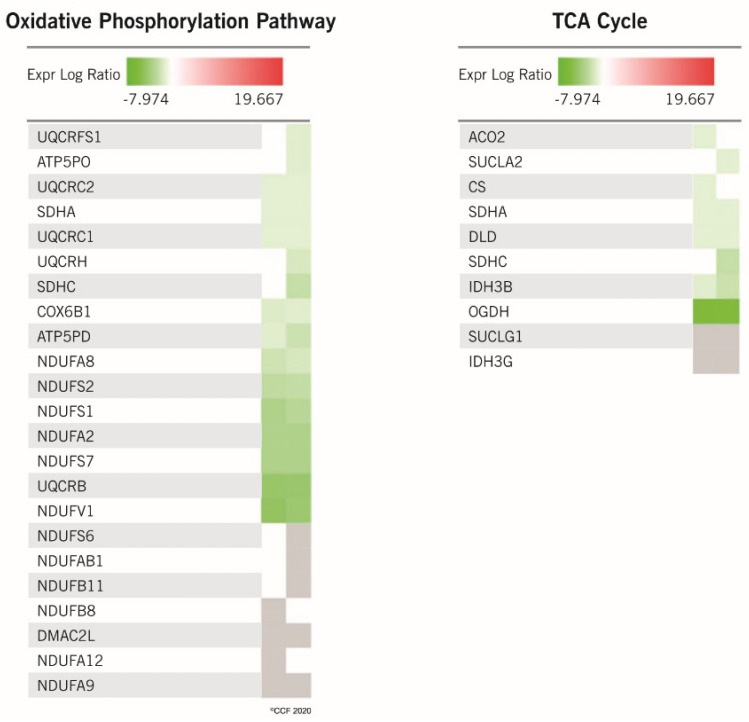
Heat map illustrating the expression of proteins associated with oxidative phosphorylation and tricarboxylic acid (TCA) cycle. Intensity of the color corresponds to the expression level of the proteins.

A bioinformatic analysis revealed that DEPs identified in the cancer group compared to fertile men were involved in the regulation of the top canonical pathways (Figure 3). Proteins associated with mitochondrial dysfunction, oxidative phosphorylation, and Sirtuin signaling pathways were dysregulated in cancer group in comparison to fertile men (Figure 3). Furthermore, a comparative analysis of two sets of DEPs predicted deactivation of oxidative phosphorylation and tricarboxylic acid (TCA) cycle (Table 3). A heat map analysis revealed an underexpression of proteins involved in oxidative phosphorylation and the TCA cycle (Figure 4).

### 2.4. Reproductive Pathways Dysregulated in Cancer Patients

**Figure 5:**
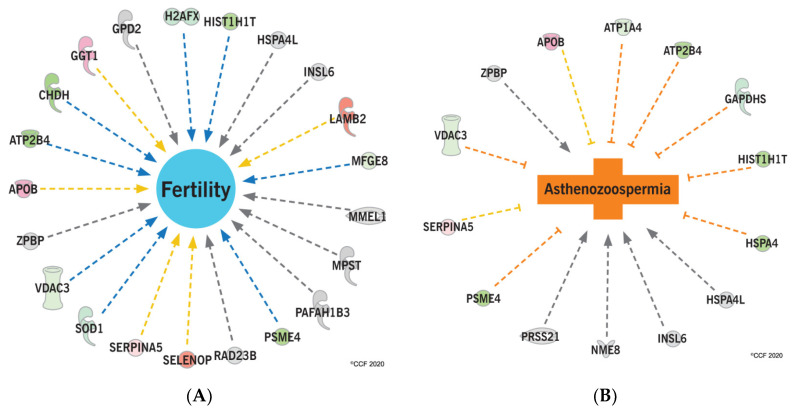
Differentially expressed proteins associated with (**A**) fertility potential and (**B**) motility of sperm in the cancer group in comparison with fertile men.

Functional analysis revealed that the upstream regulators (RICTOR, KDM5A, MAP4K4, and TRAP1) were activated due to the aberrant expression of sperm proteins in the cancer group compared to fertile men (Table 3). A pathways analysis showed that proteins associated with the sperm motility and the fertility potential were dysregulated in the cancer group (Figure 5A,B).

### 2.5. Western Blot Results

**Figure 6:**
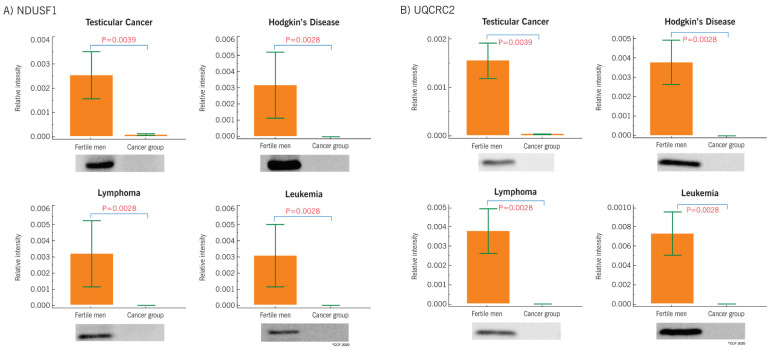

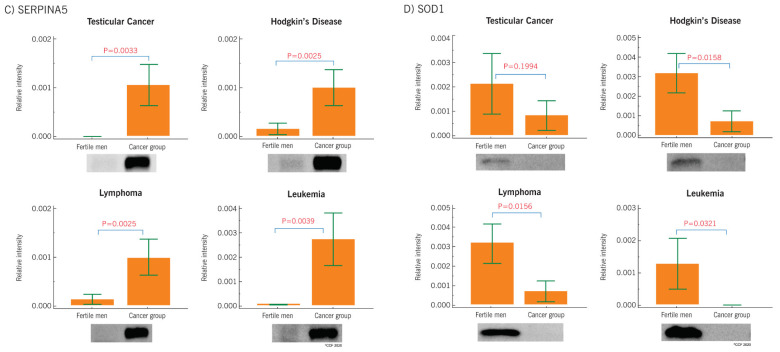
Western blot analysis of (**A**) NDUFS1 (**B**) UQCRC2, (**C**) SERPINA5 and (**D**) SOD1 in spermatozoa of cancer patients compared to fertile men.

Four key proteins, NADH:Ubiquinone oxidoreductase core subunit S1 (NDUFS1), cytochrome b-c1 complex subunit 2 (UQCRC2), SERPINA5 and superoxide dismutase 1 (SOD1), were validated using Western blot in different cancer types, such as testicular cancer, Hodgkin’s disease, leukemia, and lymphoma, in comparison with fertile men (Supplementary Figure S1). The analysis revealed the underexpression of NDUFS1 and UQCRC2 in all the cancer types compared to the fertile men (Figure 6A,B). SERPINA5 was significantly overexpressed in testicular cancer, Hodgkin’s disease, and lymphoma groups (Figure 6C), while SOD1 was underexpressed in leukemia and lymphoma cancer compared to fertile men (Figure 6D). In the case of Hodgkin’s disease and testicular cancer, SOD1 expression was comparable with fertile men (Figure 6D).

## 3. Discussion

During the past decade, the proteomics platform was used to profile the proteins in seminal fluids of patients diagnosed with several types of cancers, such as prostate cancer [25,26,27], testicular cancer [21,22,28], Hodgkin’s disease [23], and leukemia [24]. Based on their ability to detect the expression of thousands of proteins simultaneously, proteomics techniques are of great interest to unravel the molecular mechanisms leading to infertility in cancer patients. In addition, these techniques can identify putative new molecular biomarkers for diagnosis, prognosis, and therapeutics. As the cancer treatment itself can affect male fertility potential, we analyzed the sperm proteome of cancer patients before initiating any therapy, in order to understand the molecular pathways affected by the cancer disease.

Our results show an altered sperm protein profile in cancer patients. Previous proteomic studies conducted on patients before starting any therapy reported 11 and 46 DEPs to be uniquely expressed in normozoospermic testicular cancer patients [22,28], while 15 and 4 proteins were uniquely expressed in asthenozoospermic patients affected by testicular cancer [22,28], suggesting that cancer might affect sperm maturation, even if the conventional semen parameters remain unaltered. Furthermore, an overexpression of the exosomal protein, matrix metalloproteinase 9 (MMP9), has been reported in both normo- and asthenozoospermic cancer patients [28], in agreement with previous reports of its increased expression in infertile men [29]. Similarly, the proteins endothelial lipase precursor, apolipoprotein A-IV precursor, and carcinoembryonic antigen-related cell adhesion molecule 8 precursor were reported to be expressed uniquely in patients affected by Hodgkin’s disease, suggesting an impairment of metabolic processes and pathways related to the production and synthesis of reactive oxygen species (ROS) [23]. Our proteomic results support the above evidence and strongly suggest that the investigated types of cancer might be responsible for the alteration of biological pathways in sperm.

In the current study, a total of 8 proteins were identified to be uniquely expressed in cancer patients compared to fertile men. Their expression was reportedly altered in different types of cancer. Mesothelin is a glycophosphatidylinositol (GPI)-anchored protein, identified as an antigen in several human cancers affecting the pancreas, endometrium, ovary, and lung, as well as in mesothelioma and pediatric leukemia [30]. Although it is expressed in several tissues, including testis, the inactivation of mesothelin gene in experimental mice model did not affect the normal fertility, pregnancy and offspring delivery [31]. This suggests that this gene might not be crucial for reproduction. The expression of mucin isoforms has been reported in germ cells, with mucin-1 showing the highest expression in mature spermatozoa in the testis [32]. Mucin-1 is speculated to be involved in sperm maturation and transportation along the reproductive system [32]. In addition, it has been reportedly associated with survival in gastric cancer patients [33], while mucin-6 isoform was associated with favorable progression-free and cancer-specific survival in colorectal cancer [34].

Protein OS-9 involved in the ubiquitination of misfolded proteins was identified for the first time in a human osteosarcoma cell line [35,36], while desmoglein-1 was reported to interact with the testis-specific isoform of Na/K ATPase in the plasma membrane of bovine sperm during capacitation [37]. Desmoglein-1 is a component of endosomes, and it was reportedly reduced in lung cancer [38]. The hemoglobin subunit beta was highly expressed in serum of ovarian cancer patients [39], while tripeptidyl-peptidase 1 was proposed as a biomarker for colorectal and lung cancer [40,41]. Tripeptidyl-peptidase 1 is a lysosomal serine protease whose enzymatic activity has been observed in pancreatic mucinous cysts with a high probability to develop into invasive carcinoma [42]. Moreover, it has been also reported to be overexpressed in seminal plasma of patients with improved semen quality after varicocelectomy, in comparison with those who did not show any improvement [43]. Hornerin, a member of the S100 calcium-binding protein family, has been associated with the progression and poor prognosis of hepatocellular and breast cancer [44,45]. Increased expression of alpha-2-antiplasmin, a serine protease inhibitor, has been reported following varicocelectomy and in malignant ovarian cancer [46,47]. In addition, it has also been proposed as a serum biomarker for the early diagnosis of B-cell acute lymphoblastic leukemia [48]. The identification of these proteins in spermatozoa suggests that common molecular mechanisms are affected in different cancer conditions, supporting the use of these proteins as biomarkers in biological fluids other than serum such as seminal fluid.

The differential expression pattern between cancer and control groups indicate that these proteins could be directly involved in infertility-related pathways, suggesting their possible role as new mediators of male infertility in cancer patients. Apart from understanding the function of specific proteins, it is equally important to study the collective role of proteins associated with specific molecular pathways. In the current study, molecular functions associated with mitochondria were dysregulated in the spermatozoa of cancer patients, mainly due to the aberrant expression of the mitochondrial proteins. Earlier proteomic studies reported that sperm mitochondrial proteins were affected in cancer conditions such as testicular cancer [22,28] and Hodgkin’s disease [23]. Based on our proteomic results, the oxidative phosphorylation, a process linked to mitochondrial function, was defective in the spermatozoa of cancer patients (irrespective of the type of cancer included in this study). Moreover, heat map analysis revealed dysregulation of a cluster of proteins involved in oxidative phosphorylation and TCA cycle in cancer patients compared to fertile men. Western blot validation of NDUFS1 and UQCRC2 protein expression in cancer patients were in accordance with the proteomic results. NDUFS1 is a mitochondrial membrane protein and a component of Complex I which mediates the transfer of electrons to the respiratory chain. NDUFS1 has been reported to be under the regulation of RICTOR in testicular cancer patients [22]. In the present study, we noticed the underexpression of NDUFS1 and the activation of upstream regulator RICTOR, which is also responsible for the maintenance of the blood-testis-barrier [49] and the regulation of spermatogenesis [50]. This was in agreement with previous reports, showing a lower expression of NDUFS1 in the sperm of patients affected by non-seminoma testicular cancer before starting any therapy [21]. Therefore, underexpression of NDUFS1 suggests that mitochondrial function is compromised in cancer patients, with a direct effect on the fertilizing capacity of spermatozoa. Similarly, the expression of another key protein, UQCRC2, was regulated by the RICTOR [22]. UQCRC2 is reportedly involved in the TCA cycle and its underexpression has been correlated with poor fertilization rates [51] and male infertility conditions, such as varicocele [52]. In the present study, UQCRC2 was underexpressed in cancer patients, indicating multiple protein dysregulations in the mitochondria of spermatozoa regardless of the type of cancer, as demonstrated by Western blot analysis. The fertility potential of spermatozoa depends on the molecular regulation of several pathways. In the current study, several DEPs identified in the spermatozoa of cancer patients can affect the normal sperm physiological functions, especially motility or fertilizing ability.

Western blot analysis revealed an underexpression of SOD1 in Hodgkin’s disease, leukemia and lymphoma cancer groups. SOD1 is an enzyme involved in the antioxidant defense mechanism during a state of oxidative stress [53], hence, underexpression of SOD1 suggests a dysfunctional mechanism to counteract oxidative stress in cancer patients. Furthermore, SERPINA5, the protein responsible for the binding and penetration of spermatozoa into the oocyte [54], was overexpressed in spermatozoa of men with all the cancer types, suggesting an impairment of fertilization-associated pathways.

The current sperm proteome of cancer patients clearly shows that their reproductive functions are disturbed at a molecular level at the time of diagnosis. This highlights that these patients can be potentially at risk of infertility way before treatment. However, there are a few limitations of this study. The inclusion criteria did not include the stage of cancer. Furthermore, tracking the clinical outcome of our fertile controls or cancer patients post-treatment is impractical, as these subjects were not actively planning to start a family. Our study offers a snapshot of the proteome in cancer, without the possibility to identify specific molecular pathways differentially altered during the cancer progression. Moreover, the proteomics analysis was conducted on cryopreserved samples. While it is well-known that cryopreservation can reduce semen quality [13], we cannot exclude the influence of the freezing/thawing processes on the sperm proteome, which has been previously suggested by Wang et al., who observed a differential expression of proteins involved in physiological sperm pathways following cryopreservation [55]. Nevertheless, our study offers preliminary data that lays the foundation for future studies, as screening of the sperm proteome from cancer patients before treatment may help in the identification of molecular factors dysregulated in the spermatozoa of those patients.

## 4. Materials and Methods

### 4.1. Study Population

This study was approved by the Institutional Review Board (IRB) of the Cleveland Clinic Foundation. Consent was obtained from cancer patients to use their samples for research purposes. Patients affected by the following types of cancer were included in this study, regardless of the stage or specific subtype of the disease before start of any cancer therapy: testicular cancer (*n* = 40), Hodgkin’s disease (*n* = 32), lymphoma (*n* = 20) and leukemia (*n* = 17). Additionally, proven fertile and healthy donors (*n* = 19) were included as a control group. All the proteomics experiments were carried out according to the Minimum Information about a Proteomics Experiment (MIAPE) guidelines released by the Human Proteome Organization’s Proteomics Standard Initiative (HUPO-PSI) [56].

### 4.2. Semen Analysis

Semen samples were collected by masturbation after a period of sexual abstinence (2–3 days). An aliquot (6 μL) of liquefied semen was placed on a Leja sperm counting chamber (Spectrum Technologies, Healdsburg, CA, USA) and analyzed before cryopreservation, according to the WHO guidelines [57].

### 4.3. Cryopreservation and Thawing

Semen samples were cryopreserved using the TEST-Yolk Buffer (TYB, Irvine Scientific, Santa Ana, CA, USA). Aliquots of TYB equal to 25% of the sample volume were added to the specimen at room temperature, and mixed gently for 5 min using the Hema-Tek aliquot mixer (Miles Scientific, Elkhart, IN, USA). The procedure was repeated to a final 1:1 (*v*/*v*) ratio of the freezing medium to the sample. Further, the samples were dispensed into cryovials (1.5 mL; Corning, Pittsburg, PA, USA) and transferred to the freezer (−20 °C) for 8 min (static cooling), and subsequently to liquid nitrogen vapor (−80 °C) for 2 h (vapor-phase cooling). Finally, the cryovials were stored in liquid nitrogen at −196 °C until use for proteomics analysis.

### 4.4. Sperm Protein Extraction, Quantification, and Separation

Cryopreserved samples were thawed at 37 °C. The samples were then centrifuged at 4000× *g* for 7 min to remove all cryoprotective medium. Further, the samples were washed thrice with 1X PBS (phosphate-buffered saline) to remove the remnants of cryoprotectant. RIPA buffer (Sigma-Aldrich, St. Louis, MO, USA) containing Protease Inhibitor Cocktail, cOmpleteTM ULTRA Tablets, EDTA-free (Roche, Mannheim, Germany) was added to the sperm pellet (100 µL/10^6^ spermatozoa), and subsequently stored overnight at 4 °C to allow for complete lysis of the cells. After centrifugation (10,000× *g*, 30 min, 4 °C), the supernatant was transferred into a new micro-centrifuge tube. The protein content was then quantified using the bicinchoninic acid assay (BCA assay, Waltham, MA, USA).

Proteomics analyses were carried out on groups of pooled samples. Each group consisted of 3 pooled samples created by obtaining the equal contribution of sperm from each cancer type. Sperm proteins extracted from each pooled sample were mixed with SDS-PAGE buffer and run in triplicates on 1D-SDS PAGE.

### 4.5. Liquid Chromatography-Tandem Mass Spectrometer Analysis (LC-MS/MS)

The bands from each lane were excised from the gel with a gel punch. Subsequently, the bands were washed and destained in 50% acetonitrile containing 5% acetic acid, followed by dehydration in the speed-vac concentrator. The dried gel pieces were digested overnight with trypsin (5 μL of 10 ng/μL) in 50 mM ammonium bicarbonate at 37 °C. Peptides were extracted from the polyacrylamide gel in two 30 μL volumes of 50% acetonitrile and 5% formic acid, and combined and evaporated in a speed-vac concentrator to reduce the volume to 10 μL. Finally, the samples were resuspended in 15% acetic acid to make a final volume of ~30 μL for LC-MS/MS analysis.

The mass spectrometer used in this study was a Finnigan Orbitrap Elite hybrid trap mass spectrometer. Five μL volumes of the extracted peptide samples were injected into a Dionex 15 cm × 75 µm id Acclaim Pepmap C18C18 reversed-phase capillary chromatography column for LC separation, before introduction into the on-line mass spectrometer. The peptides eluted from the column by an acetonitrile/0.1% formic acid gradient at a flow rate of 0.3 μL/min were introduced into the source of the mass spectrometer on-line. The micro-electrospray ion source was set to 2.5 kV. The resulting digest was analyzed using the data-dependent multitask capability of the instrument, acquiring full scan mass spectra to determine peptide molecular weights and product ion spectra to determine the amino acid sequence in successive instrument scans.

### 4.6. Proteomic Data Analysis

The data were analyzed using all collision-induced dissociation spectra collected in the experiment to search the National Center for Biotechnology Information (NCBI) human reference sequence database with the search programs MASCOT (Mascot version 2.7; Matrix Science, Boston, MA, USA) and Sequest (Sequest version 1.4, ThermoScientific, San Jose, CA, USA) to detect the proteins present in the in-gel digestions. The results from both search programs were uploaded into the Scaffold (version 4.0.6.1, Proteome Software, Portland, OR, USA). Protein abundance was sorted as very low, low, medium, and high, depending on the number of spectral counts (SpC), while the DEPs were categorized as underexpressed, overexpressed or uniquely expressed in a particular group, based on the normalized spectral abundance factor (NSAF). Depending on the protein abundance, different *p*-values were taken into consideration: (a) very low abundance: SpC = 1.7–7; *p* ≤ 0.001; NSAF ratio ≥ 2.5 for overexpressed, ≤0.4 for underexpressed proteins; (b) low abundance: SpC = 8–19; *p* ≤ 0.01; NSAF ratio ≥ 2.5 for overexpressed, ≤0.4 for underexpressed proteins; (c) medium abundance: SpC = 20–79; *p* ≤ 0.05; NSAF ratio ≥ 2.0 for overexpressed, ≤0.5 for underexpressed proteins; (d) high abundance: SpC > 80; *p* ≤ 0.05; NSAF ratio ≥ 1.5 for overexpressed, ≤0.67 for underexpressed proteins.

A functional bioinformatics analysis was performed with the help of the publicly available annotations (Gene Ontology—GO, from GO Term Finder and GO Term Mapper), UNIPROT, STRAP and the proprietary software Ingenuity Pathway Analysis (version 2018-2019, IPA, Qiagen, Redwood City, CA, USA), to identify the differentially regulated processes, pathways, cellular distribution, and protein-protein interactions amongst proteins in the experimental and control groups, as well as for data integration.

### 4.7. Western Blotting

In this study, the following proteins were selected for validation by Western blotting, as they showed a moderate/high abundance in at least one of the groups, and were involved in reproductive functions as well as in the canonical pathways predicted to be affected in the spermatozoa of cancer patients: NDUFS1, UQCRC2, SERPINA5 and SOD1. Their differential expression was validated in different samples (*n* = 12 for each group) from those analyzed by LC-MS/MS. A total of 20 µg of proteins was fractionated through 10% SDS–polyacrylamide gels and transferred to polyvinylidene difluoride (PVDF) membranes (GE Healthcare, Marlborough, MA, USA) using the Trans-Blot Cell system (Bio-Rad Inc., Hercules, CA, USA). After transfer, nitrocellulose membranes were blocked for 1 h with 5% milk in 0.1% Tris-buffered saline containing 0.1% Tween (TBST), and incubated with primary antibodies overnight (Supplementary Table S1). The resulting blots were probed with horseradish peroxidase-conjugated secondary antibody for 2 h at room temperature. Membranes were washed four times with TBST after the primary and secondary incubations. Proteins were detected using the ECL substrate (GE Healthcare, Marlborough, MA, USA) on film and quantified using Image LabTM software (Bio-Rad Inc., Hercules, CA, USA).

### 4.8. Statistical Analysis

Statistical analysis was carried out by using the MedCalc Statistical Software version v19.0.3 (MedCalc Software bvba, Ostend, Belgium). Variable distribution was evaluated by applying the Kolmogorov–Smirnov test. The Mann–Whitney test was conducted to compare Western blotting results, while the Spearman’s rank correlation test was applied to analyze the associations. *p* value was significant when <0.05.

## 5. Conclusions

Our study shows that the cellular pathways associated with oxidative phosphorylation and the TCA cycle are affected in the spermatozoa of cancer patients. Moreover, altered expression of proteins (NDUFS1, SOD1, SERPINA5, and UQCRC2) involved in sperm fertility potential and motility suggests that the fertility of cancer patients may be at risk due to the aberrant expression of critical sperm proteins.

## Figures and Tables

**Table 1:** Semen parameters of different types of cancer patients compared with fertile donors.

Subjects	Sperm Concentration (10^6^/mL)	Total Motility (%)
Fertile Donors (*n* = 19)	81 (70.4–107.1)	71 (56.5–70.4)
Testicular cancer (*n* = 40)	16 ^a^ (9.9–29.4)	51 ^a^ (35–67.5)
Hodgkin’s disease (*n* = 32)	26.8 ^a^ (13–62)	60.5 (41.0–74.0)
Leukemia (*n* = 17)	64.7 ^a^ (43–72)	70 (33.8–95.7)
Lymphoma (*n* = 20)	55.9 ^a^ (42.4–64.7)	43.5 ^a^ (24.4–55.5)

Data are reported as median (25th–75th percentile). ^a^ *p* < 0.05 when compared to fertile donors.

**Table 2:** Sperm proteins uniquely expressed in the cancer group compared to fertile men.

S.N	UniProt ID	Protein	Abundance
Cancer vs. Fertile Men			
1.	Q13421	mesothelin isoform X1	L
2.	Q6W4 × 9	mucin-6 (MUC6) isoform X1, partial	L
3.	Q13438	protein OS-9 isoform X1	L
4.	Q02413	desmoglein-1 preproprotein	VL
5.	P68871	hemoglobin subunit beta	VL
6.	O14773	tripeptidyl-peptidase 1 preproprotein	VL
7.	Q86YZ3	hornerin	VL
8.	P08697	alpha-2-antiplasmin isoform X6	VL

L: low; VL: very low.

**Table 3:** Deactivated pathways and upstream regulators affected in spermatozoa of cancer patient.

Pathways	Z Score *
Oxidative Phosphorylation	−3.46
Tricarboxylic acid (TCA) cycle II	−2.45
Fatty acid β-oxidation I	−2.00
Glycolysis I	−2.24
**Upstream regulators**	
RICTOR	4.785
KDM5A	3.464
MAP4K4	3.162
TRAP1	2.433

* Activation or inactivation of pathways are indicated by Z score. A Z score > 2 indicates activation while a value <−2 signifies deactivation of the corresponding pathway.

## Supplementary Materials

The following are available online at https://www.mdpi.com/1422-0067/21/18/6754/s1, Supplementary Table S1: List of primary and secondary antibodies used in this study. Supplementary Table S2: List of the differentially expressed proteins identified by the bioinformatic analysis when comparing the sperm proteome cancer group with fertile men (control).

## Abbreviations

DEPs	Differentially expressed proteins
LC-MS/MS	Liquid chromatography-tandem mass spectrometer analysis
NSAF	Normalized spectral abundance factor
SpC	Spectral counts
TCA	Tricarboxylic acid cycle
