# Supplementary material for: Dysregulation of Key Proteins Associated with Sperm Motility and Fertility Potential in Cancer Patients
Source: Int J Mol Sci. 2020 Sep 15;21(18):6754. doi: 10.3390/ijms21186754 (PMC7554694; doi:10.3390/ijms21186754)
Supplement: Supplementary file 1 [file ijms-21-06754-s001.zip › ijms-884747_Supplementary Files/ijms-884747-Table S1.docx]

**Supplementary Table 1:** List of primary and secondary antibodies used in this study

| **Primary** | | | | | **Secondary** | | | |
| --- | --- | --- | --- | --- | --- | --- | --- | --- |
| **Protein** | **Antibody** | **Source** | **Manufacturer** | **Dilution** | **Antibody** | **Source** | **Manufacturer** | **Dilution** |
| NDUFS1 | Anti-Human Rabbit IgG | Rabbit monoclonal | ab157221 | 1:10000 | Anti-Rabbit  Goat IgG | Goat polyclonal | ab97051 | 1:10000 |
| SOD1 |  |  | ab 51254 | 1:20000 |  |  |  |  |
| SERPINA5 | Anti-Human Mouse IgG | mouse polyclonal | ab172060 | 1:500 | Anti-Mouse  Rabbit IgG | Rabbit polyclonal | ab6728 | 1:10000 |
| UQCRC2 |  |  | ab110411 | 1:1000 |  |  |  |  |
