# Supplementary material for: Dysregulation of Key Proteins Associated with Sperm Motility and Fertility Potential in Cancer Patients
Source: Int J Mol Sci. 2020 Sep 15;21(18):6754. doi: 10.3390/ijms21186754 (PMC7554694; doi:10.3390/ijms21186754)
Supplement: Supplementary file 1 [file ijms-21-06754-s001.zip › ijms-884747_Supplementary Files/Western Blot Images.pdf]

Western Blot Images

Figure S1: Western blot of NDUFS1 in spermatozoa of men with testicular cancer compared to fertile men.

Figure S2: Western blot of NDUFS1 in spermatozoa of men with hodgkin's disease compared to fertile men.

Figure S3: Western blot of NDUFS1 in spermatozoa of men with lymphoma compared to fertile men.

Figure S4: Western blot of NDUFS1 in spermatozoa of men with leukemia compared to fertile men.

Figure S5: Western blot of UQCRC2 in spermatozoa of men with testicular cancer compared to fertile men.

Figure S6: Western blot of UQCRC2 in spermatozoa of men with hodgkin's disease compared to fertile men.

Figure S7: Western blot of UQCRC2 in spermatozoa of men with lymphoma compared to fertile men.

Figure S8: Western blot of UQCRC2 in spermatozoa of men with leukemia compared to fertile men.

Figure S9: Western blot of SERPINA5 in spermatozoa of men with testicular cancer compared to fertile men.

Figure S10: Western blot of SERPINA5 in spermatozoa of men with hodgkin's disease compared to fertile men.

Figure S11: Western blot of SERPINA5 in spermatozoa of men with lymphoma compared to fertile men.

Figure S12: Western blot of SERPINA5 in spermatozoa of men with leukemia compared to fertile men.

Figure S13: Western blot of SOD1 in spermatozoa of men with testicular cancer compared to fertile men.

Figure S14: Western blot of SOD1 in spermatozoa of men with hodgkin's disease compared to fertile men.

Figure S15: Western blot of SOD1 in spermatozoa of men with lymphoma compared to fertile men.

Figure S16: Western blot of SOD1 in spermatozoa of men with leukemia compared to fertile men.

Figure S1:

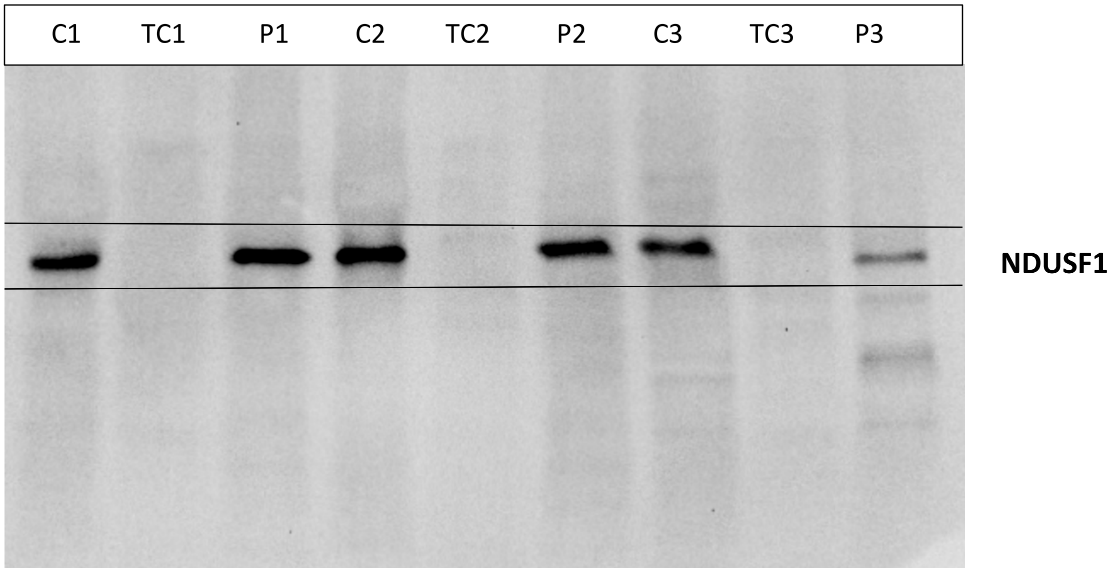

C: control group with fertile men, TC: testicular cancer, P: infertile men

Note: Expression profile of proteins from control group and testicular cancer group were only used for comparison in the current study.

Figure S2:

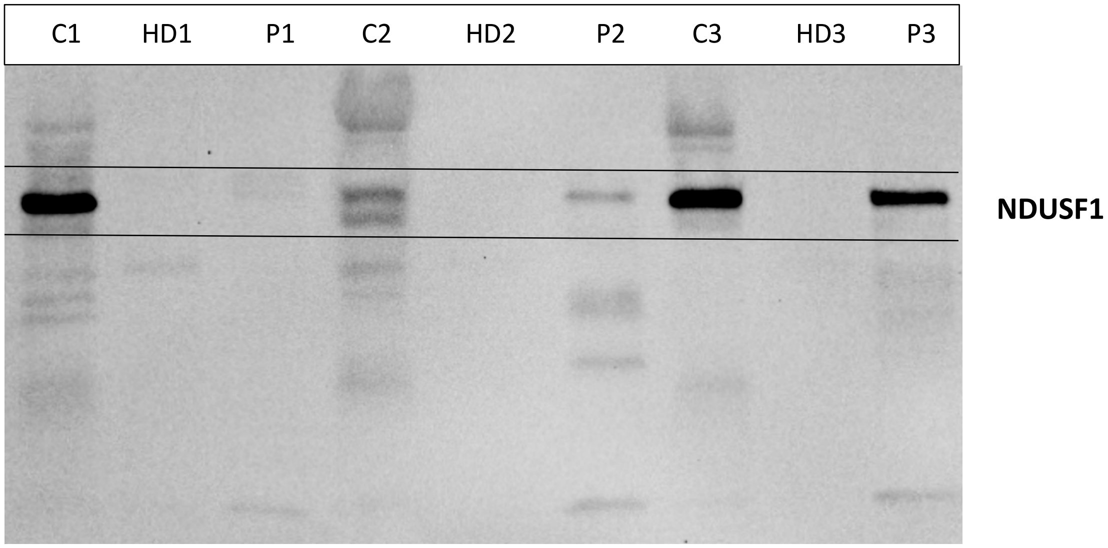

C: control group with fertile men, HD: hodgkin's disease, P: infertile men

Note: Expression profile of proteins from control group and hodgkin's disease group were only used for comparison in the current study.

Figure S3:

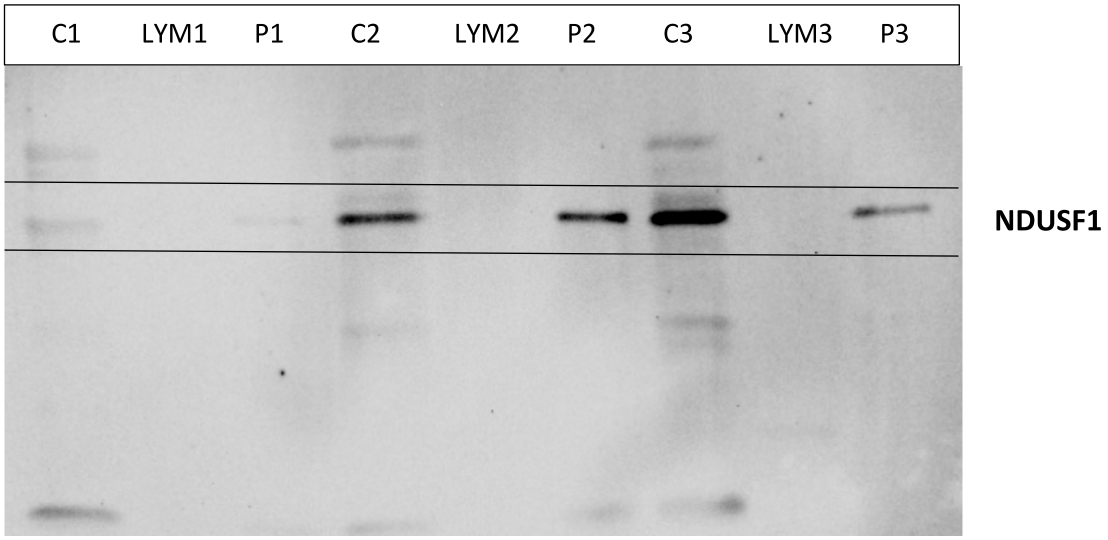

C: control group with fertile men, LYM: lymphoma, P: infertile men

Note: Expression profile of proteins from control group and lymphoma group were only used for comparison in the current study.

Figure S4:

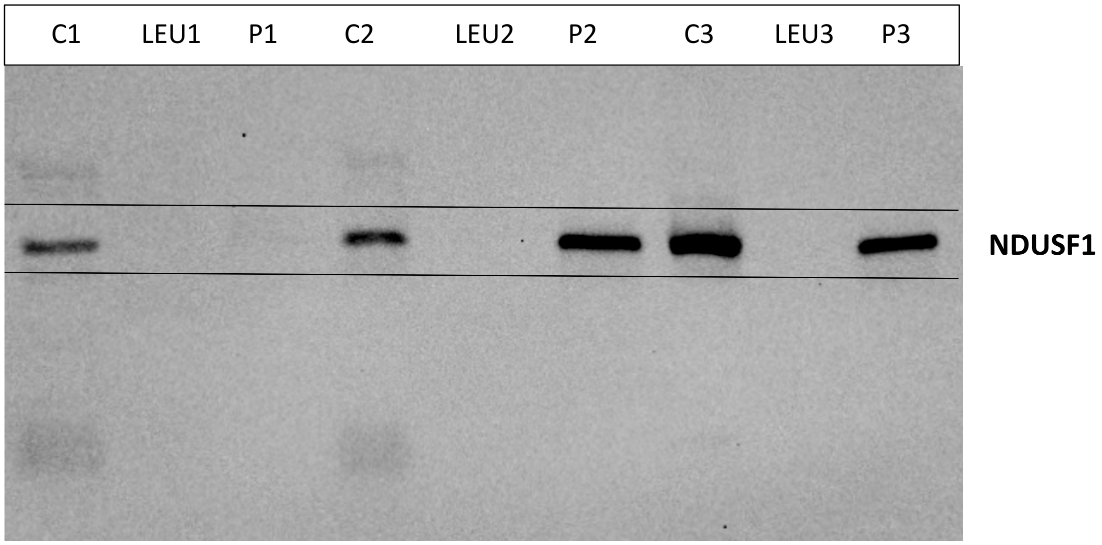

C: control group with fertile men, LEU: leukemia, P: infertile men

Note: Expression profile of proteins from control group and leukemia group were only used for comparison in the current study.

Figure S5:

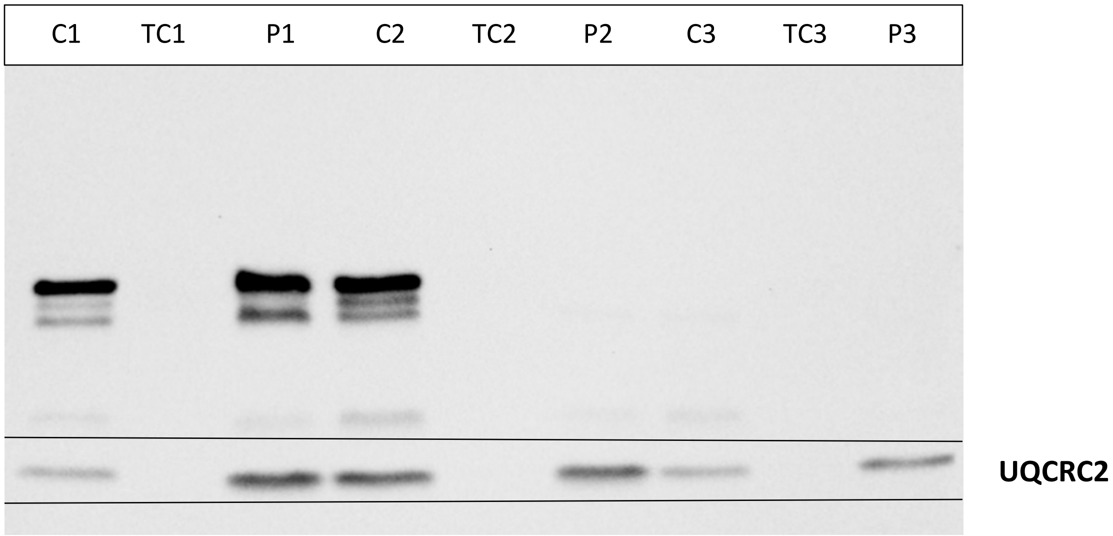

C: control group with fertile men, TC: testicular cancer, P: infertile men

Note: Expression profile of proteins from control group and testicular cancer group were only used for comparison in the current study.

Figure S6:

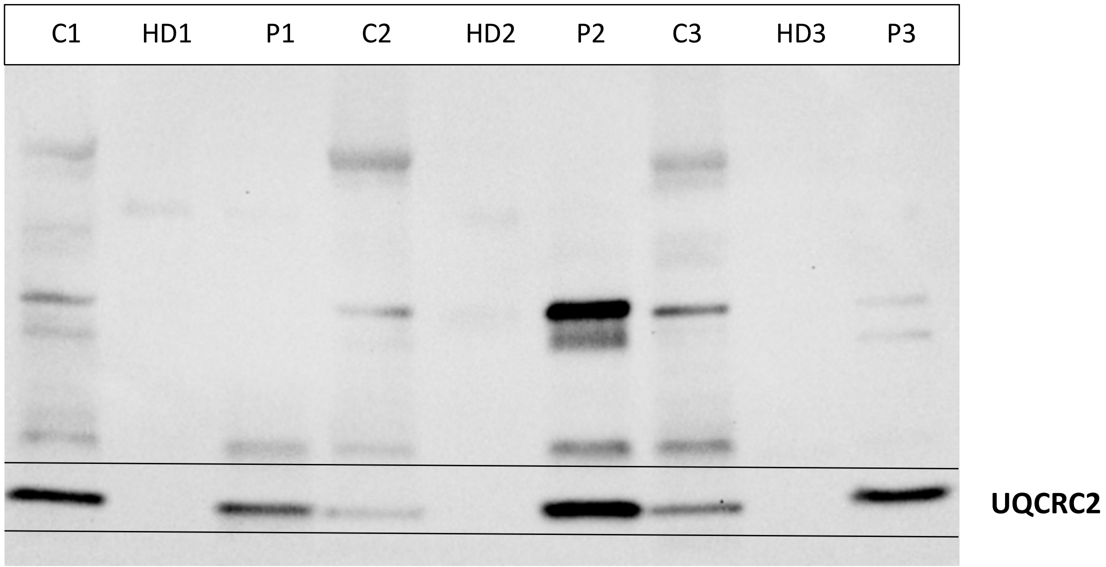

C: control group with fertile men, HD: hodgkin's disease, P: infertile men

Note: Expression profile of proteins from control group and hodgkin's disease group were only used for comparison in the current study.

Figure S7:

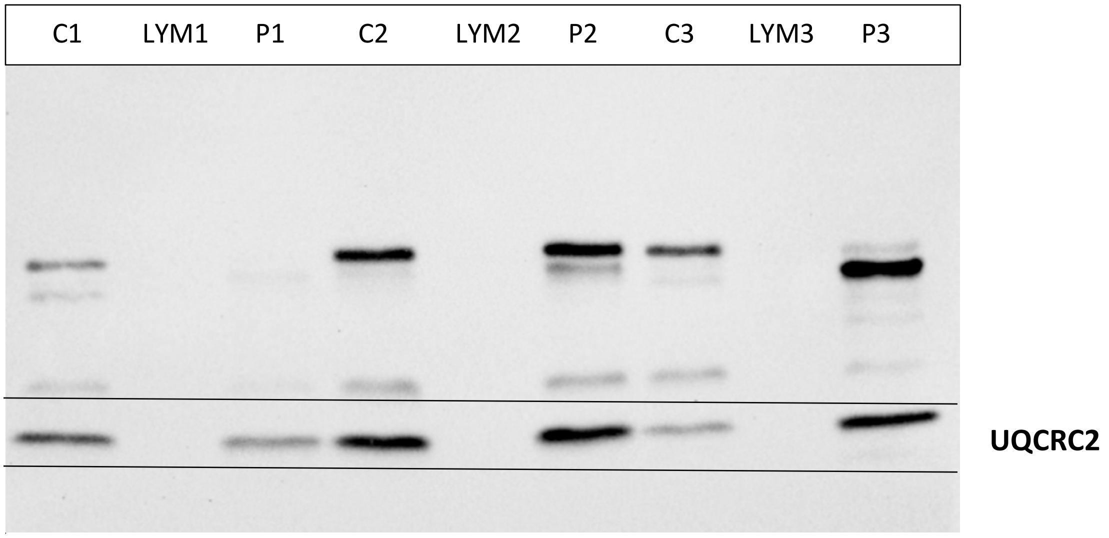

C: control group with fertile men, LYM: lymphoma, P: infertile men

Note: Expression profile of proteins from control group and lymphoma group were only used for comparison in the current study.

Figure S8:

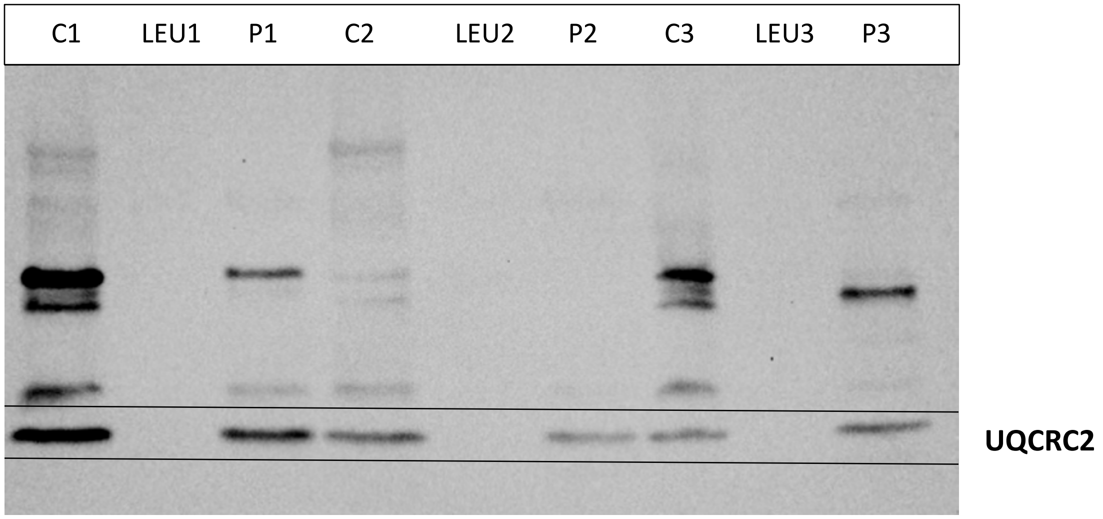

C: control group with fertile men, LEU: leukemia, P: infertile men

Note: Expression profile of proteins from control group and leukemia group were only used for comparison in the current study.

Figure S9:

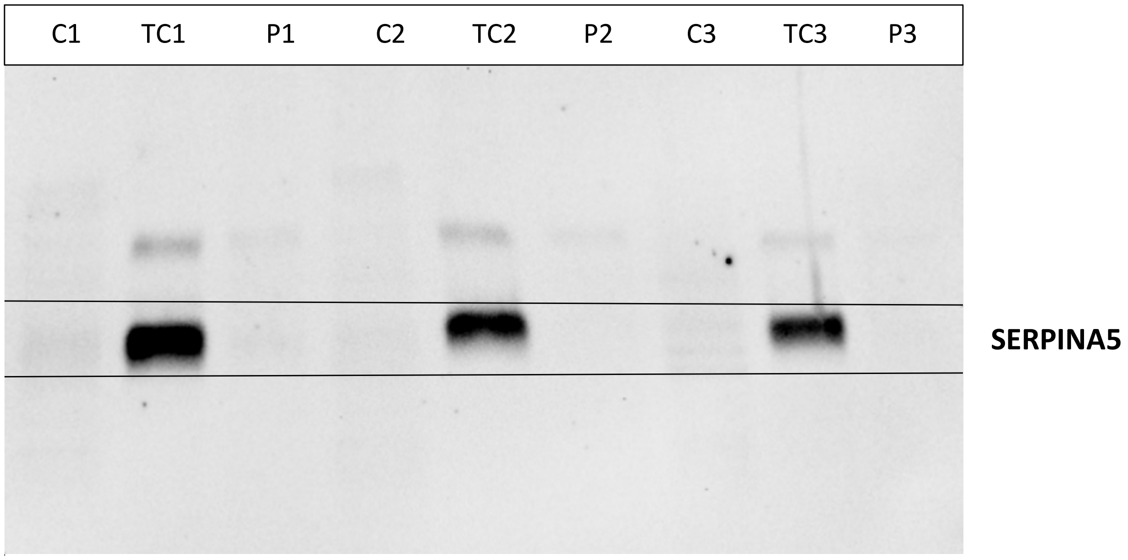

C: control group with fertile men, TC: testicular cancer, P: infertile men

Note: Expression profile of proteins from control group and testicular cancer group were only used for comparison in the current study.

Figure S10:

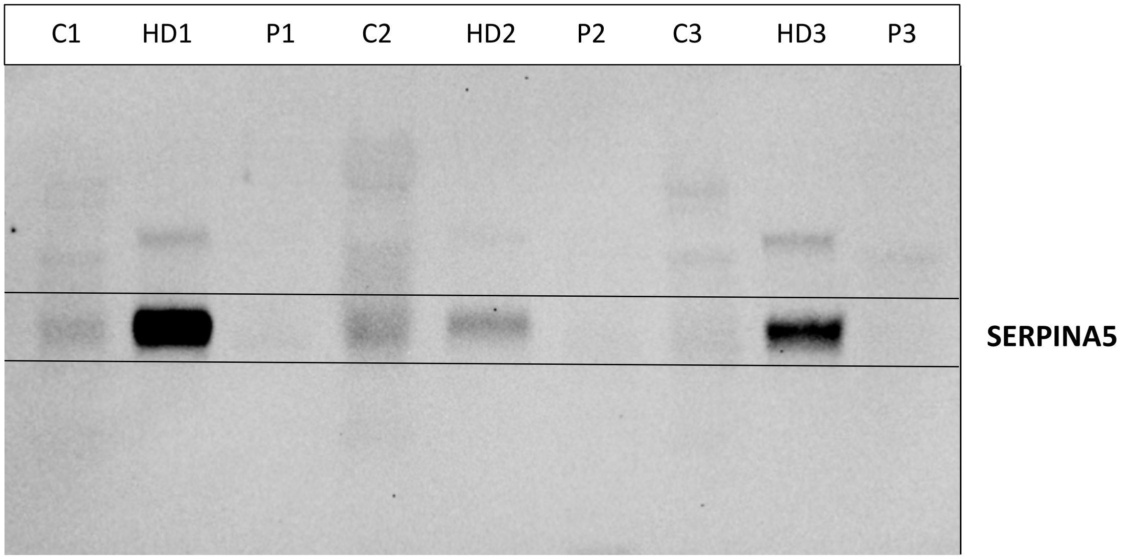

C: control group with fertile men, HD: hodgkin's disease, P: infertile men

Note: Expression profile of proteins from control group and hodgkin's disease group were only used for comparison in the current study.

Figure S11:

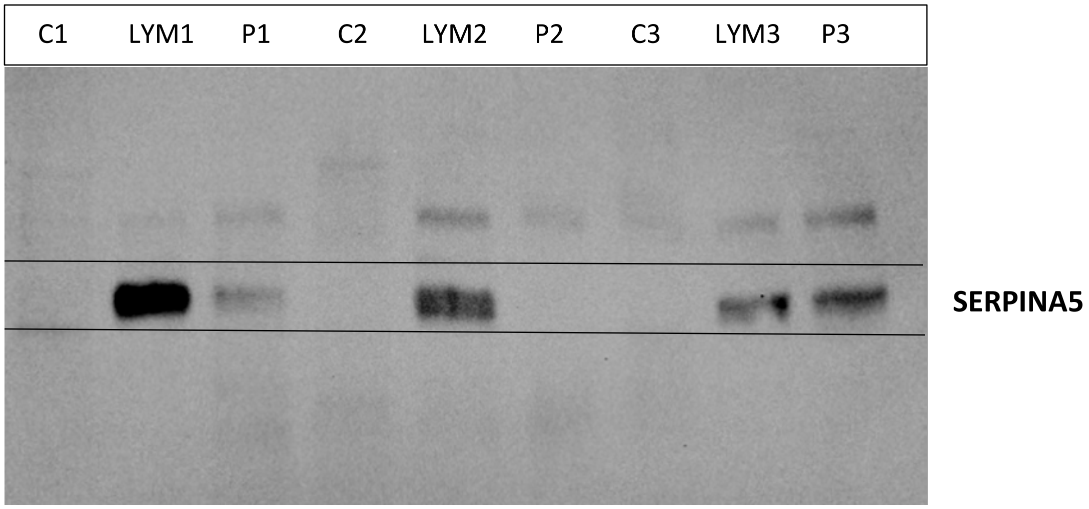

C: control group with fertile men, LYM: lymphoma, P: infertile men

Note: Expression profile of proteins from control group and lymphoma group were only used for comparison in the current study.

Figure S12:

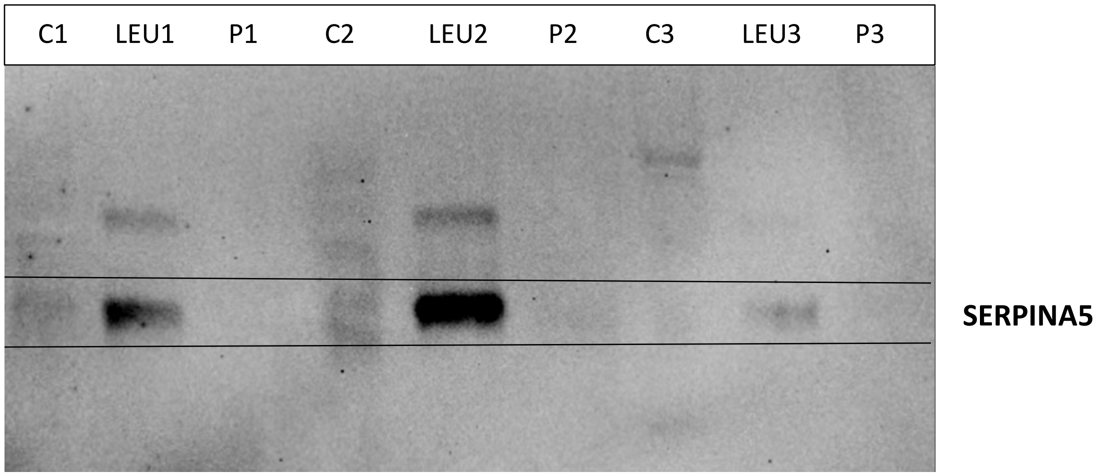

C: control group with fertile men, LEU: leukemia, P: infertile men

Note: Expression profile of proteins from control group and leukemia group were only used for comparison in the current study.

Figure S13:

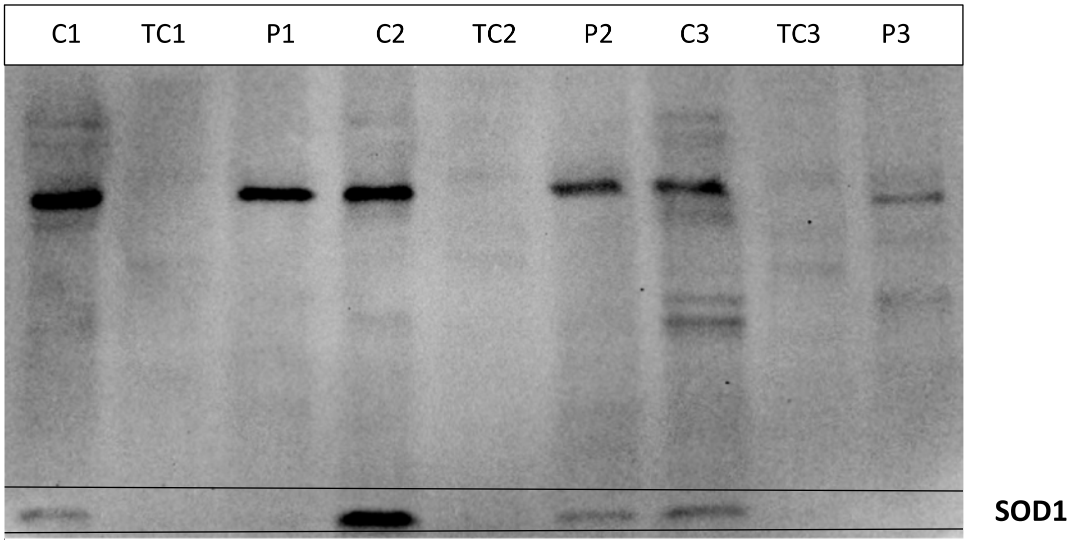

C: control group with fertile men, TC: testicular cancer, P: infertile men

Note: Expression profile of proteins from control group and testicular cancer group were only used for comparison in the current study.

Figure S14:

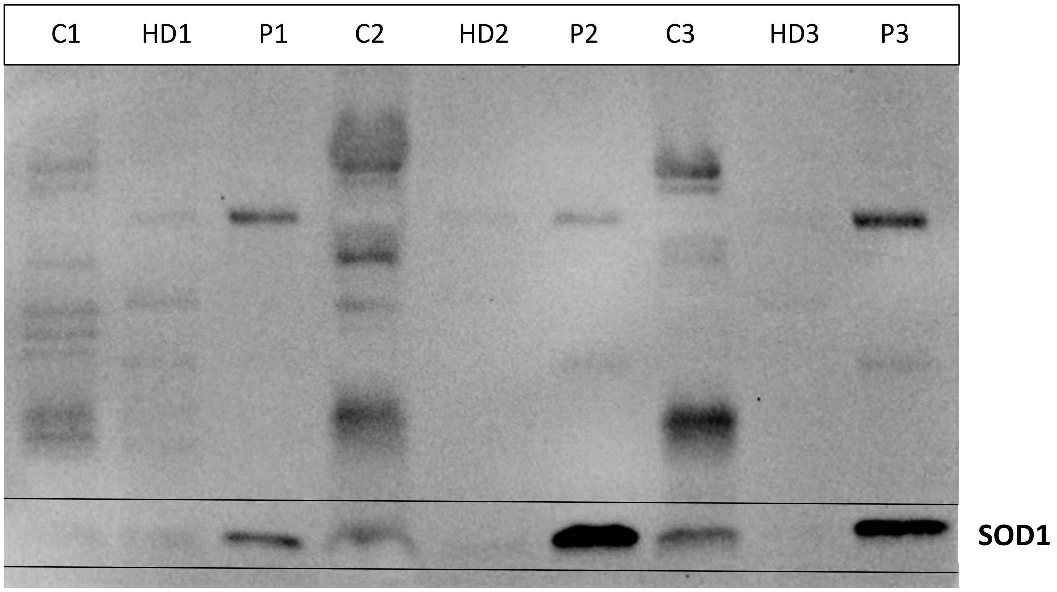

C: control group with fertile men, HD: hodgkin's disease, P: infertile men

Note: Expression profile of proteins from control group and hodgkin's disease group were only used for comparison in the current study.

Figure S15:

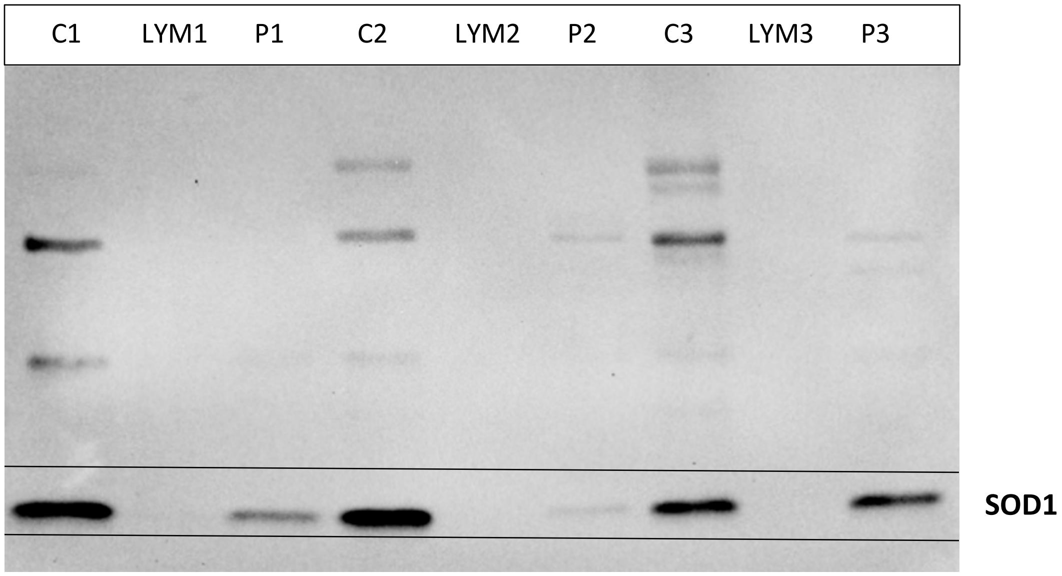

C: control group with fertile men, LYM: lymphoma, P: infertile men

Note: Expression profile of proteins from control group and lymphoma group were only used for comparison in the current study.

Figure S16:

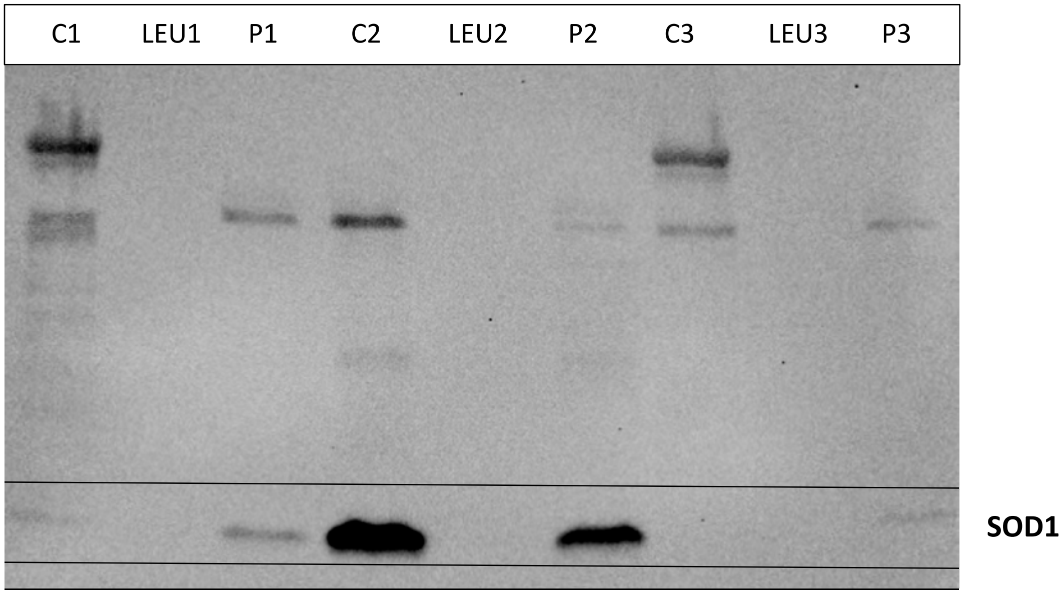

C: control group with fertile men, LEU: leukemia, P: infertile men

Note: Expression profile of proteins from control group and leukemia group were only used for comparison in the current study.
